# Aberrant Activation of the RANK Signaling Receptor Induces Murine Salivary Gland Tumors

**DOI:** 10.1371/journal.pone.0128467

**Published:** 2015-06-10

**Authors:** Maria M. Szwarc, Ramakrishna Kommagani, Allison P. Jacob, William C. Dougall, Michael M. Ittmann, John P. Lydon

**Affiliations:** 1 Department of Molecular Cellular Biology, Baylor College of Medicine, One Baylor Plaza, Houston, Texas, United States of America; 2 Therapeutic Innovation Unit (TIU), Amgen Inc, Seattle, Washington, United States of America; 3 Department of Pathology and Immunology, Baylor College of Medicine, and Michael E. DeBakey VAMC, Houston, Texas, United States of America; Charles P. Darby Children's Research Institute, UNITED STATES

## Abstract

Unlike cancers of related exocrine tissues such as the mammary and prostate gland, diagnosis and treatment of aggressive salivary gland malignancies have not markedly advanced in decades. Effective clinical management of malignant salivary gland cancers is undercut by our limited knowledge concerning the key molecular signals that underpin the etiopathogenesis of this rare and heterogeneous head and neck cancer. Without knowledge of the critical signals that drive salivary gland tumorigenesis, tumor vulnerabilities cannot be exploited that allow for targeted molecular therapies. This knowledge insufficiency is further exacerbated by a paucity of preclinical mouse models (as compared to other cancer fields) with which to both study salivary gland pathobiology and test novel intervention strategies. Using a mouse transgenic approach, we demonstrate that deregulation of the Receptor Activator of NFkB Ligand (RANKL)/RANK signaling axis results in rapid tumor development in all three major salivary glands. In line with its established role in other exocrine gland cancers (*i*.*e*., breast cancer), the RANKL/RANK signaling axis elicits an aggressive salivary gland tumor phenotype both at the histologic and molecular level. Despite the ability of this cytokine signaling axis to drive advanced stage disease within a short latency period, early blockade of RANKL/RANK signaling markedly attenuates the development of malignant salivary gland neoplasms. Together, our findings have uncovered a tumorigenic role for RANKL/RANK in the salivary gland and suggest that targeting this pathway may represent a novel therapeutic intervention approach in the prevention and/or treatment of this understudied head and neck cancer.

## Introduction

Representing ~3–6% of head and neck cancers (or an annual incidence of 2–3 cases per 100,000 people in the USA) [1], malignant salivary gland tumors are relatively rare and markedly heterogeneous in terms of histologic types and subtypes, presenting formidable clinical challenges for diagnosis and treatment [2, 3]. Unfortunately, attempts to more effectively diagnose and treat these malignant salivary gland neoplasms have been stymied by our current limited knowledge of the key molecular signals that underpin the initiation, progression, recurrence, and/or metastasis of this tumor type. Also, the salivary gland cancer field severely lacks preclinical animal models (as compared to other cancer fields, such as breast cancer) with which to both study the molecular pathogenesis of this head and neck cancer and test new or re-purposed anti-neoplastic targeted therapies that may improve patient outcome.

Encoded by the *TNFSFII* gene in the human, Receptor Activator of NFkB Ligand (RANKL; also known as osteoprotegrin ligand, osteoclast differentiation factor, and tumor necrosis factor (TNF)-related activation-induced cytokine) is a member of the TNF superfamily of cytokines and exclusively signals *via* its receptor, RANK (encoded by the *TNFSFRIIA* gene); reviewed in [4]. Binding of RANKL and RANK occurs through their respective transmembrane homotrimers. However, RANKL has multiple forms including a transmembrane protein, a primary secreted form and a soluble form resulting from enzymatic cleavage. The secreted form can arise from either metalloproteinase cleavage or alternative splicing. Through paracrine and/or autocrine pathways, the RANKL/RANK signal governs a remarkable array of signaling cascades that underpin a broad spectrum of physiological processes, ranging from osteoclastogenesis and bone homeostasis, dental eruption, T cell activation and dendritic cell survival, promotion of immunotolerance, secondary lymphoid organogenesis, medullary thymic epithelial development, microfold (M) cell differentiation from intestinal cells in Peyer’s patches, central whole-body thermomodulation, hair renewal and epidermal growth in the hair follicle, to mammary gland morphogenesis; reviewed in [4, 5].

In keeping with its pleiotropic role in normal physiology, deregulation of the RANKL/RANK signaling axis underlies the initiation and/or progression of numerous pathophysiologies, including rheumatoid arthritis (an autoimmune disorder) [6], postmenopausal osteoporosis [7], and rare familial bone pathologies (*i*.*e*. Paget’s disease) [8]. Overlapping its role in osteoimmunopathologies, the RANKL/RANK signaling axis is essential for malignant progression of many diverse cancers—multiple myeloma [9], non-small cell lung cancer[10], renal cell carcinomas [11], prostate [12, 13], and breast cancer [14–19]—to a metastatic state. As a pro-metastatic factor for many of these cancers, RANKL/RANK signaling promotes primary tumor cell proliferation, invasion, migration, and colonization by influencing a wide spectrum of cellular properties from driving the epithelial-mesenchymal transition (EMT) program [18, 20, 21], expanding the cancer stem cell population [18, 22], attracting infiltrating tumor associated macrophages to the tumor microenvironment [23, 24], to facilitating metastatic colonization of circulating tumor cells to distant anatomic sites such as bone and lung [11, 12, 14, 17, 18, 23–25].

Here, we provide a brief report which describes rapid salivary gland tumor development and progression following the unscheduled activation of the RANKL/RANK signaling axis in a transgenic mouse model. Aberrant RANKL/RANK signaling reprograms the salivary gland epithelium to an aggressive proliferative phenotype that leads to the emergence of multifocal palpable salivary gland tumors within a short latency period with histopathologic features consistent with a poorly differentiated mucoepidermoid cancer [26, 27]. These findings highlight this cytokine signaling pathway both as a new molecular mediator of salivary gland tumorigenesis and a potential target for intervention. Indeed, the ability to significantly attenuate salivary tumor progression using a targeted RANKL intervention strategy, suggests a possible new avenue for therapeutic prevention and/or treatment in the future. Considering our current knowledge gap concerning the molecular mechanisms that underlie salivary gland tumorigenesis along with the increasing support for the RANKL/RANK signaling axis in the promotion of other head and neck cancers and inflammatory disorders [28–30], we believe our studies offer a new conceptual framework with which to gain further molecular insight into the etiopathogenesis of this understudied cancer which may lead to novel clinical avenues for diagnosis, prognosis, and/or treatment in the future.

## Materials and Methods

### Mice and salivary gland tumor studies

Under a 12h light: 12h dark recurrent photocycle in a temperature controlled environment (22°C ± 2°C), mice were housed in an AAALAC fully accredited *vivarium* under the auspices of the Center for Comparative Medicine at Baylor College of Medicine. Unless otherwise stated, mice received irradiated Teklad global soy protein-free extruded rodent diet (Harlan Laboratories Inc., Indianapolis, IN) along with fresh water *ad libitum*. Animal experiments were conducted in accordance with the guidelines set forth by the Guide for the Care and Use of Laboratory Animals as published by the National Research Council (Eighth Edition 2011). Animal protocols used in these studies were approved by the Institutional Animal Care and Use Committee (IACUC) at Baylor College of Medicine under animal protocol numbers: AN-1513 and AN-544. Animal experiments have been approved by IACUC. Anesthesia: ketamine 37.5 mg, xylazine 1.9 mg and acepromazine 0.37 mg sq to 5 ml with 2.45 ml sterile water given at 0.75–1.5 ml/kg BW, IP. Euthanasia was conducted by cervical disarticulation while under surgical plane of anesthesia. CO2 euthanasia was conducted by use of automated CO2 euthanasia chambers (EUTHANEX).

The generation and initial characterization of the mouse mammary tumor virus (MMTV)-RANKL transgenic (TG) mouse was previously described [15]. Briefly, these TG mice carry multiple tandem copies of a transgene in which expression of murine RANKL is driven by the MMTV long terminal repeat (LTR) composite promoter. Since the establishment of F_1_ lines from at least three separate founders (F_0_), TG lines (hemizygous for the transgene) have been continuously back-crossed with the FVB/NJ wild type (WT) mouse strain (stock number: 001800; The Jackson Laboratory, Bar Harbor, Maine) for at least 15 generations. For tumor incidence studies, female mice were monitored at least once-a-week during the initial period when tumors were not yet detectable. Mice were monitored daily when a visual or palpable salivary gland tumor was evident. Mice were euthanized when palpable salivary gland tumors reached ~1.5cm at the longest diameter; tumor latency, number (or tumor load), size, and location were recorded for each mouse.

The MTB, TZA and MTB/TZA mice have been described previously [31]. Briefly, the MTB mouse is an effector transgenic mouse in which the MMTV-LTR promoter drives expression of the reverse tetracycline-dependent transactivator (rtTA). The TZA responder transgenic mouse carries the TetO-LacZ reporter transgene in which the expression of the beta-galactosidase (β-gal or LacZ) reporter gene is driven by a tetracycline-responsive promoter comprised of the human cytomegalovirus early promoter linked to tet operator sequences. The MTB/TZA bigenic mouse was generated by crossing MTB and TZA transgenic mice (both in a FVB/N background strain). Doxycycline in the food and water induces transgene-derived β-gal expression in MTB/TZA tissues in which the MMTV-LTR is active (*i*.*e*. the mammary and salivary gland [31]). For these experiments, bigenic test and monogenic control mice were provided grain-based sterile green ½” pellets fortified with doxycycline at 200mg/kg (Bioserv, St. Louis, MO) as well as water (supplied in light-protected bottles) containing doxycycline at 2mg/ml (BD Clontech, San Diego, CA) supplemented with 5% sucrose (Fisher, Pittsburgh, PA) to ameliorate taste aversion. Induction potency was maintained by changing the doxycycline treated water every 3–4 days. For these studies, previously described protocols for detecting β-gal activity (X-gal staining) in tissue sections were followed [32].

### Treatment with RANK-Fc

The RANK-Fc reagent (lot #: p9995.26) was kindly provided by Amgen Inc. for these studies; RANK-Fc was dissolved in sterile phosphate buffered saline (PBS) pH 7.0 for injection purposes. Acting as a soluble recombinant RANKL antagonist [33], RANK-Fc comprises the murine extracellular domain of RANK (amino acids: 1 through Pro 213) fused to the Fc portion of human immunoglobulin G1 (IgG1). For the RANK-Fc treatment experiment, 6-week-old TG mice received in the intrascapular region a subcutaneous (s.c.) injection of RANK-Fc (10mg/kg body weight) three times per week for 2–months. Corresponding control treatment groups (WT and TG mice) received 100μl of PBS (vehicle) by s.c. injection at a similar frequency and duration. After 2-months of treatment, mice were euthanized before their salivary gland tissue was carefully prosected, weighed, and processed further for histological and molecular analysis.

### Histochemical Analysis

Histopathology of TG salivary gland tumors were assessed according to the histologic criteria detailed by the World Health Organization [26, 27]. To histologically score cells in S-phase of the cell-division cycle, mice were administered 5-bromo-2’-deoxyuridine (BrdU (1mg per 20g body weight); Amersham Biosciences, Piscataway, NJ) by intraperitoneal (i.p.) injection two hours before euthanasia. Tissue fixation, processing, paraffin-embedding, and microtome sectioning have been previously reported [15]. Primary antibodies for the following antigens used in these studies were: RANK ((AF692) 1:200 dilution; R&D Systems, Inc., Minneapolis, MN); RANKL ((AF462) 1:200 dilution; R&D Systems, Inc.); p63 ((SC-8431)1:600 dilution; Santa Cruz Biotechnology Inc., Dallas, TX); vimentin ((ab11256) 1:50 dilution; Abcam Inc., Cambridge, MA); snail/slug ((ab63371) 1:500; Abcam Inc. (note: this antibody detects both snail and slug which have similar molecular weights (26–29 kDa)); PCNA ((ab2426) 1:200 dilution; Abcam Inc.); zeb1 ((ab87280) 1:200 dilution; Abcam Inc,); and BrdU ((ab1893) 1:150 dilution; Abcam Inc.). Following primary antibody incubation, sections were incubated with the appropriate biotinylated secondary antibody (1:200 dilution; Vector Laboratories, Burlingame, CA) for 1h followed by incubation with Vectastain Elite horseradish peroxidase conjugated avidin (Vector Laboratories) for 30 minutes at room temperature. Immunopositivity was visualized using the DAB peroxidase substrate kit (Vector Laboratories) before counterstaining with Harris hematoxylin (Poly Scientific, Bay Shore, NY). Following washes with increasing concentrations of ethanol and xylene, sections were overlaid with permount (Fisher Scientific, Pittsburgh, PA) before affixing a cover slip. For dual immunofluorescence studies, established protocols were followed [15] using the following primary antibodies: sheep anti-BrdU ((ab1893) 1:100 dilution; Abcam Inc.); goat anti-mouse RankL ((AF692) 1:50 dilution; R & D Systems); rabbit anti-human PCNA ((ab2426) 1:200 dilution; Abcam Inc.) and corresponding secondary antibodies (diluted 1:200) respectively: anti-sheep IgG conjugated with alexafluor 594 ((A11016) Invitrogen Inc.); anti-goat IgG conjugated with alexafluor 488 ((A11055) Invitrogen Inc.); and anti-rabbit IgG conjugated with alexafluor 546 ((A11010) Invitrogen Inc.). Slides were mounted with Vectashield mounting medium containing 4’, 6’-diamidino-2-phenylindole (DAPI; Vector Laboratories Inc.). Images were digitally captured using a color chilled AxioCam MRc5 digital camera attached to a Carl Zeiss AxioImager A1 Upright microscope (Zeiss, Jena, Germany); digital images were processed and assembled using Photoshop CS5 (Adobe Systems Inc., San Jose, CA). To determine the percentage of salivary gland cells that is immunopositive, four TG mice and an equal number of WT were used. The average number of immunopositive cells (*i*.*e*. BrdU positive) was estimated from a total of 500 salivary gland cells from three separate salivary gland sections per mouse (four mice per genotype and treatment). Final counts were expressed as an average percentage (± standard deviation (S.D.)) of total cells counted.

### Western immunoblot analysis

Salivary gland or tumor tissue was homogenized on ice in Tris-Triton-X100 tissue lysis buffer complete with mini-protease inhibitors (Roche, Basel, Switzerland). Following homogenization, the tissue lysate was incubated on ice for 20 min before the protein supernatant was obtained following centrifugation. Before electroblotting to polyvinylidene difluoride membranes (Millipore, Billerica, MA), proteins (20μg/lane) were resolved by denaturing electrophoresis using a 4–15% gradient sodium dodecylsulfate-polyacrylamide gel (Biorad Laboratories, Hercules, CA). Non-specific IgG binding was blocked using 5% milk in Tris-buffered saline (TBS) with 0.1% Tween. The following primary antibodies (at a 1:1000 dilution) were used to detect immunoreactivity: cyclin D1 ((RB-9041) Thermo Scientific Inc.)); PCNA ((SC-56) Santa Cruz Inc.); vimentin ((3832) Cell Signaling Inc.); P63 ((ab63371) Abcam Inc.); RANKL ((ab9957) Abcam Inc.); snail/slug ((ab63371) Abcam Inc.); Zeb1 ((ab87280) Abcam Inc.); and β-actin (loading control) at 1:10000 dilution ((A1978) Sigma-Aldrich, St. Louis, MO). Immunoreactive bands were detected using the appropriate secondary antibody conjugated with horseradish peroxidase and visualized with SuperSignal West Pico Chemiluminescent Substrate (Thermo Scientific, Rockford, IL). To enable western blots to be reprobed with different antibodies, blots were stripped of primary and secondary antibodies using Restore Western Blot Stripping Buffer ((21059) Thermo Scientific Inc.).

### Statistical Analyses

Kaplan-Meier curves were analyzed with the log-rank test using GraphPad Prism 6 (GraphPad Software, Inc., La Jolla CA). Quantile comparisons were performed to inspect normality of data. Equality of variances was analyzed using the Bartlett test of homogeneity of variances. Statistical analyses were performed with Kruskal-Wallis rank sum test with post hoc analysis performed with Wilcoxon rank-sum test in R Studio with the R Commander package (R Studio Inc., Boston, MA). Multiple comparisons were adjusted with the Holm method. Non specific (n.s.) was considered p>0.05 whereas p ≤0.05 (*); p ≤0.01 (**); and p ≤0.001 (***) were considered significant.

## Results

### Murine salivary gland tumorigenesis is triggered by RANKL

**Fig 1:**
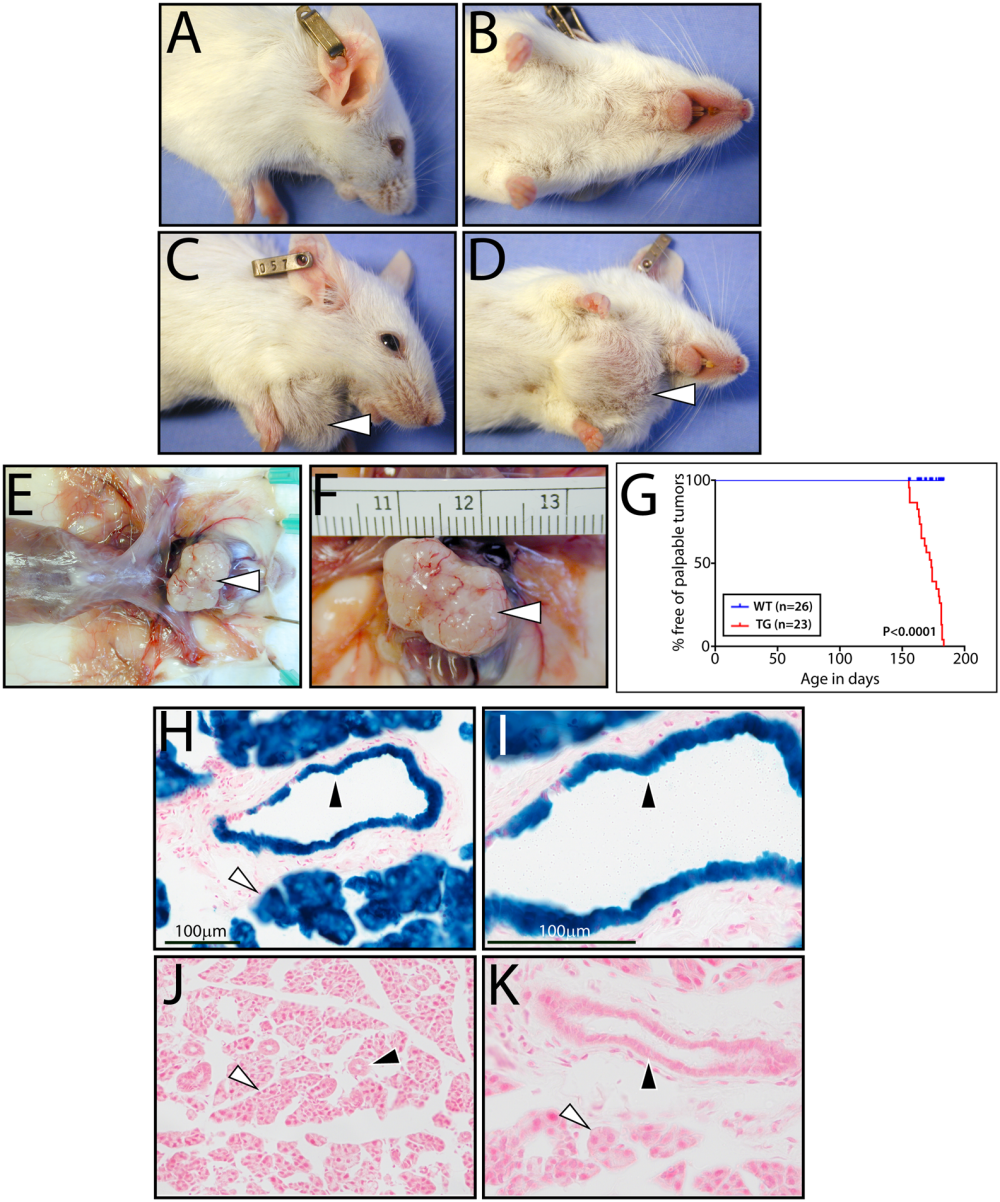
The RANKL/RANK signaling axis induces palpable salivary gland tumors in mice. (A/B) and (C/D) show lateral and ventral views of a 6-month-old WT and an aged-matched TG mouse respectively. The white arrowhead in (C) and (D) points to a palpable submandibular salivary gland tumor in the TG mouse. (E) and (F) show low and high magnification images respectively of the submandibular salivary gland tumor *in situ*. (G) The Kaplan-Meier curve plots the percentage free of palpable salivary gland tumors versus time in days for WT (blue (n = 26)) and TG (red (n = 23)) mice. (H) and (I) are low and high magnification images respectively that show detection of β-gal activity (LacZ staining) in normal salivary gland tissue from a 10-week-old MTB/TZA bigenic reporter mouse previously administered doxycycline for 1-week. Note: black and white arrowheads point to strong blue staining in the epithelium of salivary gland ducts and acinar bodies respectively. (J) and (K) are low and high magnification images respectively of similarly stained salivary gland tissue sections from a TZA monogenic control mouse which was also administered doxycycline for 1-week; scale bar in (H) and (I) apply to (J) and (K) respectively.

Our MMTV-RANKL transgenic (TG) mouse was originally generated in the FVB/Nmob strain (Jackson Labs; stock number: 001491) and exhibited palpable salivary gland tumors within 210–340 days-of-age [15]. Tumorigenesis was predominantly located in one type of salivary gland per TG mouse in which tumors were clearly demarcated from surrounding normal tissue at the histological level. Since our original report, we have crossed these TG lines into the FVB/NJ strain for 15 generations; the FVB/NJ strain is derived from the JAX Genetic Stability Program (GSP; (http://jaxmice.jax.org/strain/001800.html)) which is designed to avoid the accumulation of genetic drift that can occur over time in other strains [34, 35]. In the FVB/NJ strain, the TG mouse group developed palpable salivary gland tumors (1.5cm diameter) significantly earlier in life (155 days of age for the first mouse in the TG group to present palpable salivary gland tumors (1.5cm diameter) with a mean time of 173 days for palpable tumor emergence for the entire TG group (Fig 1A–1G)). For this study, mice were euthanized when palpable salivary gland tumors reached 1.5cm in the longest diameter; however, salivary tumors (~1cm diameter) in these mice could be detected as early as 84 days-of-age (data not shown). Importantly, all TG mice displayed palpable salivary gland tumors within a short time frame (28 days) as compared to 130 days in the FVB/Nmob strain [15]. In the case of the FVB/Nmob strain, TG mice exhibited palpable salivary gland tumors (1.5cm diameter) with a mean tumor incidence of 277 days [15] *versus* 173 days in the FVB/NJ strain. Unlike the FVB/Nmob strain [15], salivary gland tumors in the FVB/NJ strain often developed in more than one type of salivary gland (*i*.*e*. in both the submandibular and parotid salivary gland) per mouse (S1 Fig). Using the MTB/TZA bigenic reporter [31], lacZ staining clearly reveals that the MMTV promoter (used to target transgene expression) is active in epithelial cells of salivary ducts and in acinar cells (Fig 1H and 1I). These salivary gland cell types also express endogenous RANK (S2 Fig) supporting the conclusion that transgene-derived RANKL expression is targeted to salivary gland cells that express its cognate receptor. The above tumor studies were conducted with female mice; however, similar results were obtained using male mice (data not shown). Collectively, these results indicate that by crossing our original TG mouse into the FVB/NJ background strain, we have generated a markedly improved mouse model which exhibits RANKL/RANK dependent salivary gland tumors at a significantly shorter latency period and at a much higher penetrance.

### Aberrant RANKL/RANK signaling elicits significant salivary gland cell proliferation in the mouse

**Fig 2:**
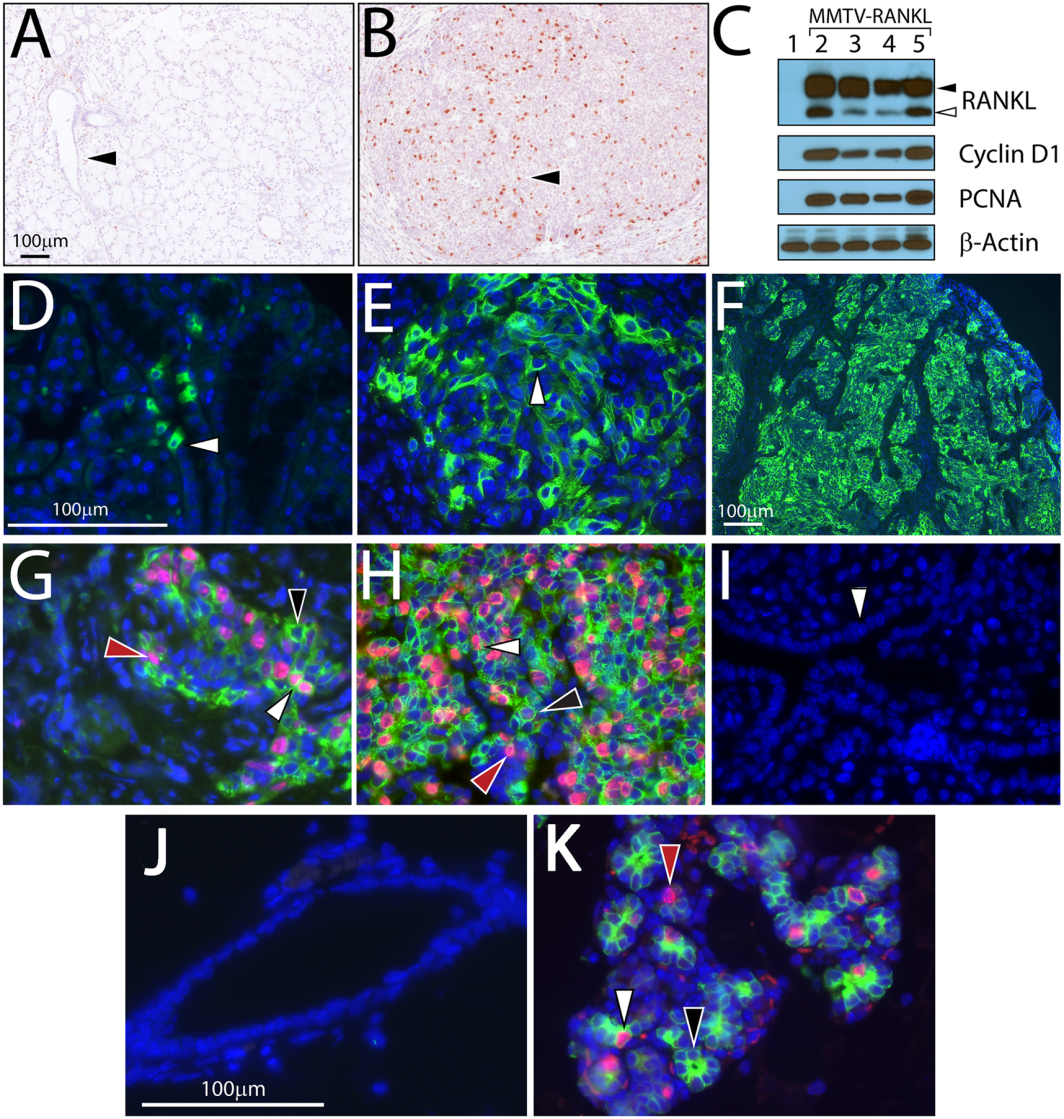
Aberrant RANKL overexpression exerts potent mitogenic effects in the murine salivary gland. (A) shows a typical histologic section of a salivary gland from a 160 day old WT mouse which was stained for BrdU incorporation (black arrowhead points to a transverse section of a salivary gland duct). (B) shows a histologic section of a palpable salivary gland tumor from an aged-matched TG mouse (the black arrowhead points to a tumor cell representative of many that are positive for BrdU incorporation); scale bar in (A) also applies to (B). (C) is a western immunoblot of salivary gland protein isolated from WT mice (lane 1) and of salivary gland tumor protein isolated from four TG mice (lanes 2–5). Western results are shown for both the transmembrane and secretory versions of RANKL (black and white arrowheads respectively) and for cyclin D1 and PCNA which represent established biomarkers for cell proliferation; β-actin serves as a protein loading control. The white arrowhead in D, E, and F shows immunofluorescence detection at the cellular level of the expansion of RANKL expression in salivary gland tissue obtained from 50, 100 and 160 day old TG mice respectively. (G) and (H) represent dual immunofluorescence detection of RANKL and PCNA positivity in salivary gland tissue obtained from 50 and 160 day old TG mice respectively; white arrowhead shows a salivary gland cell positive for both RANKL and PCNA expression whereas the black arrowhead points to a cell that is positive and negative for RANKL and PCNA expression respectively. (I) is salivary gland tissue from a 160 day old WT mouse which shows no staining for endogenous RANKL and PCNA expression (white arrowhead points to a longitudinal section of a salivary gland duct). (J) and (K) are representative images of dual immunofluorescence staining for RANKL (green) and PCNA (red) in WT and TG mammary tissue respectively. Note the absence of endogenous RANKL and PCNA expression in the WT mammary epithelium (J). In panel (K), white, black, and red arrowheads point to a mammary epithelial cell that is RANKL/PCNA, RANKL, and PCNA positive respectively. Scale bar in (D) also applies to (E); (G); (H); and (I); scale bar in (J) applies to (K).

In keeping with its potent mitogenic role in other target tissues [15–19, 25, 36, 37], RANKL drives significant salivary gland tumor cell proliferation (Fig 2A and 2B; S3 Fig) with expression levels of established biomarkers of cell division (*i*.*e*. cyclin D1 and proliferation cell nulear antigen (PCNA)) markedly elevated as compared to normal salivary gland tissue (Fig 2C). Immunofluorescence detection of transgene-derived RANKL expression at defined times of salivary gland tumor progression suggest that clonal expansion of targeted RANKL tumor cells may represent one cellular mechanism by which RANKL elicits salivary gland tumor expansion during the early stages of cancer progression (Fig 2D–2F). Note: Endogenous RANKL expression and cellular proliferation are not detected in the salivary gland of the age-matched WT mouse (Fig 2I); the absence of endogenous RANKL expression also concurs with the Western result shown in Fig 2C (lane 1)). Interestingly, dual immunofluorescence for transgene-derived RANKL and PCNA expression reveals salivary gland tumor cells which are double positive for RANKL and PCNA expression along with tumor cells which are positive for either RANKL or PCNA expression. Since RANK is expressed in most epithelial cells of the normal salivary gland (S2 Fig), these findings indicate that RANKL driven salivary tumor cell proliferation may occur by both autocrine and paracrine signaling (Fig 2G and 2H). In the case of autocrine signaling, RANKL positive cells undergo proliferation, suggesting that RANKL may licence cell cycle progression within its cell of origin. With paracrine signaling, a sub-population of RANKL negative cells expands by receiving paracrine proliferative signals from juxtaposed RANKL positive cells. Intriguingly, similar RANKL signaling has been reported in the murine mammary gland epithelium [15–17, 36–41]. Similar to the WT salivary gland (Fig 2I), the WT mammary epithelium does not express endogenous RANKL (Fig 2J); however, the TG mammary gland expresses RANKL in a similar pattern as detected in the TG salivary gland above (Fig 2K (white and black arrowheads)). In agreement with the extensive RANKL-induced mammary epithelial proliferation in Fig 2K, significant alveologenesis in the TG mammary gland is evident by whole mount analysis (S4 Fig). Together, these results demonstrate that RANKL/RANK signaling acts as a strong mitogen in the salivary gland through cellular mechanisms similar to those previously found in the mammary epithelium.

### Murine salivary gland tumors induced by RANKL/RANK signaling exhibit an aggressive tumor phenotype

**Fig 3:**
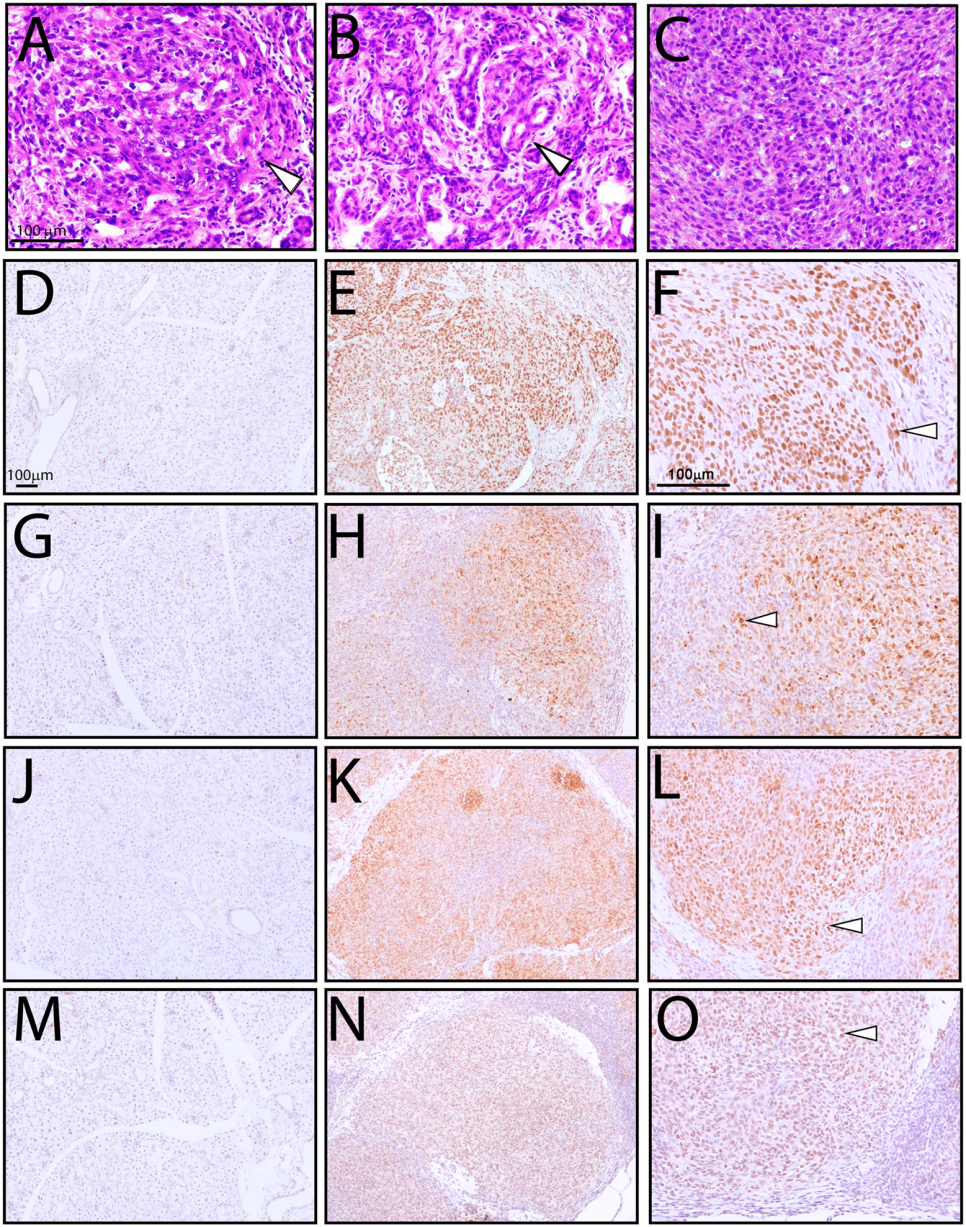
Murine salivary gland tumors induced by RANKL/RANK signaling display the molecular hallmarks of an aggressive head and neck cancer. Hematoxylin and eosin (H&E) stained sections of salivary gland tumor tissue from a 160 day old TG mouse displays: (A) squamous differentiation (white arrowhead); (B) glandular epithelium (white arrowhead); and (C) a field of intermediate cells with small amounts of cytoplasm. Scale bar in (A) applies to (B) and (C). (E/F); (H/I); (K/L) and (N/O) show immunohistochemical detection of p63; vimentin; snail/slug; and zeb1 at low and high magnifications respectively (white arrowheads point to representative tumor cells positive for one of the four markers. Note: (D (p63); G (vimentin); J (snail/slug); and M (zeb1)) are the similarly stained salivary gland tissue from age-matched WT mice. Scale bar in (D) applies to (E); (G); (H); (J); (K); (M); and (N) whereas the scale bar in (F) also applies to (I); (L); and (O).

The histopathology of these TG salivary gland tumors strongly supports an aggressive malignant phenotype. Tissue sections stained with H&E revealed a multinodular and infiltrative tumor with both focal and zonal necrosis evident. Tumors consisted predominantly of spindle cells which showed areas of brightly eosinophilic cytoplasm consistent with squamous differentiation (Fig 3A (white arrowhead)); in some areas, glands were also observed (Fig 3B (white arrowhead)). In addition, there were areas of intermediate cells with small amounts of cytoplasm (Fig 3C). These histological features indicate the tumor is a poorly differentiated mucoepidermoid carcinoma [26, 27]. Further supporting an aggressive tumor phenotype, a number of molecular markers (*i*.*e*. slug/snail; vimentin; zinc finger E-box binding homeobox 1 (zeb1); and p63) associated with advanced stage malignancies and poor prognosis were significantly elevated in these tumors (Fig 3D–3O; and western analysis in S5 Fig)). Only low levels of p63 expression was detected in the salivary gland epithelium of the age-matched WT mouse (S6 Fig).

### Treatment with RANK-Fc significantly attenuates RANKL driven murine salivary gland tumorigenesis

**Fig 4:**
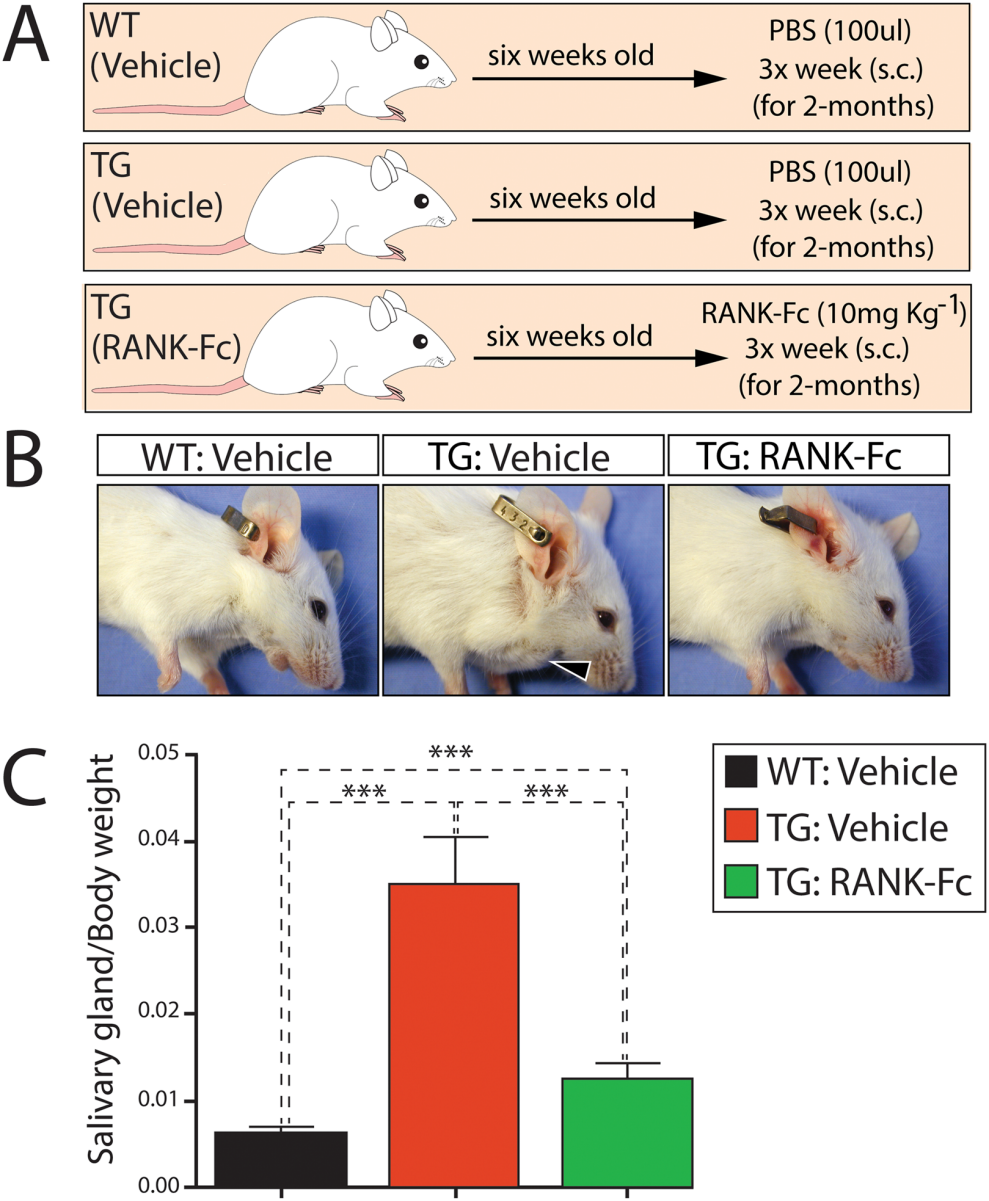
Early administration of RANK-Fc significantly attenuates RANKL dependent murine salivary gland tumorigenesis. (A) Schematic of RANK-Fc administration protocol: At 6 weeks-of-age, TG mice received a s.c. injection of RANK-Fc or vehicle (PBS) 3 times a week for 2-months; WT mice received PBS only. Mice were analyzed 2 months later. (B) Lateral views of WT and TG mice treated with vehicle as well as TG mice treated with RANK-Fc; mice were photographed two months following the end of vehicle or RANK-Fc treatment. Black arrowhead in middle panel points to the emergence of a palpable salivary gland tumor in the vehicle treated TG mouse. (C) Histogram displays the salivary gland/body weight ratio for each genotype and treatment condition (***denotes p<0.001; mean ± standard error of the mean (S.E.M.); and n = 20 mice/genotype/treatment).

Considering the advanced state of salivary gland tumorigenesis attained in these TG mice following a relatively short latency period (Fig 1), we wondered whether early therapeutic targeting of the RANKL/RANK signaling axis would effectively prevent or significantly attenuate the emergence of this tumor type. The rationale for this study is based on the urgent need for effective treatment strategies to prevent the aggressive reemergence of regressed (or dormant) human salivary gland tumors that often follows surgical resection of the primary tumor [2, 3]. Using a modification of an estabished treatment protocol [33] (Fig 4A), we found that early administration of RANK-Fc (which blocks the RANKL-RANK interaction) significantly attenuated salivary gland tumor progression in the TG mouse group (Fig 4B and 4C). The RANK-Fc treatment protocol used in these studies also significantly attenuated mammary alveologenesis, underscoring the effectiveness of this treatment protocol (S7 Fig). Because the focus of this first line of studies was to determine whether early administration of RANK-Fc can attenuate early changes in salivary gland tumor formation, mice were euthanized at 98 days-of-age before these mice exhibit palpable salivary gland tumors with a 1.5cm diameter. It should be noted that there was a small but statistically significant increase in salivary gland weight in RANK-Fc treated TG mice compared to WT siblings. Although a topic for future investigation, this weight difference may indicate that RANKL dependent salivary gland tumors can adapt early to a RANKL independent growth program in which a small subset of RANKL negative salivary gland cells which proliferate in response to the paracrine signal(s) from RANKL positive cells expand without further requirement for RANKL. Irrespective of the above, these initial studies provide “proof-of-concept” that therapeutically targeting the RANKL/RANK signaling axis may offer a new treatment modality in the clinical management of salivary gland cancers.

## Discussion

In this short report, we demonstrate through transgenesis that unscheduled activation of the endogenous RANK signaling receptor reprograms the ductal and acinar epithelium of the murine salivary gland to an aggressive tumor phenotype which leads to advanced stage malignancies within a short latency period. Although RANKL overexpression is targeted to the epithelium of both the salivary and mammary gland of these mice, palpable tumors are only detected in the salivary gland in these studies, suggesting that the epithelium of this exocrine tissue is highly responsive to the RANKL/RANK signal. Histologically, the tumors are poorly differentiated mucoepidermoid cancers which are known aggressive neoplasms in humans [26, 27]. In addition, there was marked elevation in expression levels of established biomarkers of EMT (*i*.*e*. zeb1; vimentin; snail; and slug) parallels similar cellular and molecular changes by which aberrant RANK signaling accelerates the progression of cancers to advanced stage disease [12, 18, 20, 21, 25, 42]. Noteworthy, members of the snail/slug and zeb families of E-box binding transcription factors and their targets (*i*.*e*. vimentin) are established molecular activators of the epithelial-mesenchymal transition (EMT) program [43–45]. Essential to tumor progression, activation of the EMT program not only confers immotile epithelial cells with a migratory and invasive mesenchymal phenotype but enables these cells to avoid apoptosis and senescence and to acquire cancer stem cell-like properties [46, 47]. Overexpression of the p63 transcription factor, which exerts critical roles in the development of basal-type tumors and stem cell renewal [48, 49], further supports the advanced progressive state of this cancer. Importantly, these findings are consistent with the established involvement of RANKL/RANK signaling in the acquisition of these aggressive malignant features in other tumors, such as cancers of the mammary and prostate gland [12, 18, 20, 21, 25, 42, 50, 51].

Recent investigations demonstrate that enhanced RANK signaling in the untransformed human breast MCF-10A epithelial cell line induces EMT through increased expression of vimentin, zeb1 and 2, snail, and slug [18]. These studies also revealed that increased RANK signaling elicits other cellular changes associated with transformation and cancer stemness, such as increased cell motility, expansion of the cancer stem cell population (*i*.*e*. CD44(+); CD24(-)), and anchorage-independent growth. Whether similar cellular changes are employed by the RANKL/RANK signaling axis during salivary gland tumorigenesis—as observed in tumor cells of the mammary gland [18, 21, 25, 42, 50, 52] as well as of the prostate gland [12, 20, 53], kidney [11], and liver [51]—is a research question to be addressed by future studies. Pertinent to this study, however, RANKL/RANK signaling has been shown to elicit the EMT process to advance tumor progression in other head and neck cancer subtypes, such as oral carcinomas [54–56]. Although aberrant expression of RANKL in human salivary gland neoplasms—as modeled in our transgenic mouse—has yet to be evaluated, there is compelling support for an important role for deregulated RANKL/RANK signaling in general human oral pathobiology. Apart from driving the EMT program, RANKL is secreted from oral squamous cell carcinomas to support osteolytic invasion of the mandibular bone [29, 30, 57–59], reminiscent of metastasis by other RANKL dependent cancers.

While constitutive activation of the RANK signaling receptor results in the development of salivary gland tumors within a short period, early treatment with RANK-Fc significantly attenuates tumor emergence. This first line of studies provides “proof-of concept” that therapeutic silencing of the RANKL/RANK pathway may represent a plausible molecular targeting approach (either alone or in combination with other targeted therapies) for the treatment of primary salivary gland tumors and for the prevention of secondary tumor recurrence and metastasis that frequently manifests following surgical resection. This concept is made all the more plausible by the regulatory approval for the immunotherapeutic use of denosumab; reviewed in [60]. A human IgG2 monoclonal antibody, denosumab specifically binds to both soluble and membrane bound primate RANKL thereby preventing this cytokine from binding its signaling receptor; note, RANK-Fc is a murine mimetic of denosumab. Currently, denosumab (Prolia) is used in the treatment of postmenopausal osteoporosis, and accelerated osteopenia due to hormone ablation therapy for prostate and breast cancer, and (under the trade name of Xgevia) bone metastasis from a number of solid tumors and non-resectable giant cell tumors; reviewed in [60]. However, due to the equally important extraskeletal roles of RANKL/RANK signaling; reviewed in [5], preclinical investigations in particular have highlighted this signaling axis as an attractive target for therapeutic intervention in pathologies other than adverse bone resorption: from cancers of the mammary gland [61] to the oral cavity [62, 63]. In the context of these ongoing studies, our studies reported here underscore the importance of investigating further whether this cytokine signaling axis represents a molecular vulnerability that could be exploited in the treatment of salivary gland cancers.

In conclusion, our first line of mouse transgenic studies highlights a critical role for the RANKL/RANK signaling cue in the etiopathogenesis of aggressive salivary gland tumors. Apart from providing histologic and molecular insight into the ontogenesis of this understudied exocrine cancer, this preclinical animal model will be invaluable for testing new preventative and tumoricidal treatment modalities, an urgent imperative if we are to significantly improve the current status of clinical management of this elusive head and neck cancer.

## Supporting Information

S1 Fig The RANKL/RANK signaling axis induces palpable tumors in all major salivary glands. (A) is a lateral view of an age-matched WT mouse; (B-N) are representative images of lateral and ventral views of TG mice showing palpable salivary gland tumors (white arrowhead). Palpable tumors were detected in the parotid (B; H; J; and L), submandibular (D; F; H; and N), or both salivary gland types (B and H).
(TIF)

S2 Fig Expression of RANK in the murine salivary gland. (A) shows typical RANK expression in the epithelium of salivary gland ducts and acini (white arrowheads); salivary gland cells that are negative for RANK expression are indicated by black arrowheads. (B) shows no immunoreactive staining in the absence of the primary antibody to RANK; scale bar in (A) also applies to (B). (C) and (D) are higher magnification images of regions shown in (A); scale bar in (C) also applies to (D).
(TIF)

S3 Fig The RANKL/RANK signaling axis significantly drives murine salivary gland tumor proliferation. (A) and (C) are low and high magnification images respectively of salivary gland tissue from WT mice stained for BrdU incorporation; white arrowheads point to salivary gland ducts. (B) and (D) are low and high magnification images respectively of salivary gland tumor tissue from age-matched TG mice similarly stained for BrdU incorporation; black arrowheads point to BrdU positive tumor cells. Scale bar in (A) and (C) apply to (B) and (D) respectively. (E) Histogram displaying the average percentage of epithelial cells (± standard deviation (S.D.)) that are positive for BrdU incorporation in salivary gland tumors and salivary gland tissues of TG and WT mice respectively (*denotes p<0.05 (n = 4 mice/genotype)).
(TIF)

S4 Fig The RANKL/RANK signaling axis induces significant ductal side-branching and alveologenesis in the mammary gland of the TG virgin mouse. (A) and (B) are high magnification images of mammary gland whole mounts from age-matched WT and TG mice respectively. (B) Note the extensive ductal side-branching and alveologenesis in the TG mammary gland. Scale bar in (A) applies to (B).
(TIF)

S5 Fig RANKL induces EMT markers in murine salivary gland tumors. Western immunoblot result of salivary gland and salivary gland tumor protein isolated from WT mice (lane 1) and from four TG mice (lanes 2–5) respectively. Note the striking elevated levels of Zeb1, p63, vimentin, and snail/slug which represent common EMT molecular traits of an aggressive cancer phenotype.
(TIF)

S6 Fig High magnification images of p63, snail/slug, zeb1, and vimentin expression in salivary gland tissue of WT mice. (A-C) are low and high magnification images of immunohistochemical detection of low levels of p63 expression in a subset of basal cells of the salivary gland epithelium of WT mice (black arrowhead). Snail/slug (D-F) and vimentin (G and H) are not detected whereas low levels of zeb1 expression are detected in a few cells per field (black arrowhead). Scale bar in (A) also applies to (D), (G), and (I); scale bar in (B) also applies to (C), (E), (F), (H) and (J).
(TIF)

S7 Fig Mammary gland alveologenesis is suppressed by RANK-Fc treatment. (A) Whole mount of mammary gland derived from a WT mouse treated with vehicle (PBS); see Fig 4 for full details of treatment protocol. (B) Whole mount of mammary gland from a TG mouse treated with vehicle. Note: clear evidence of epithelial alveologenesis driven by transgene-derived RANKL (black arrowhead). (C) Whole mount of mammary gland from a TG mouse following treatment with RANK-Fc. Note the absence of alveologenesis with RANK-Fc treatment. Scale bar in panel (A) applies to all panels.
(TIF)

S1 File Scans of whole western blot films from Fig 2C. (PDF)

S2 File Scans of whole western blot films from S5 Fig. (PDF)
