# Supplementary figures and images for: Aberrant Activation of the RANK Signaling Receptor Induces Murine Salivary Gland Tumors
Source: PLoS One. 2015 Jun 10;10(6):e0128467. doi: 10.1371/journal.pone.0128467 (PMC4464738; doi:10.1371/journal.pone.0128467)

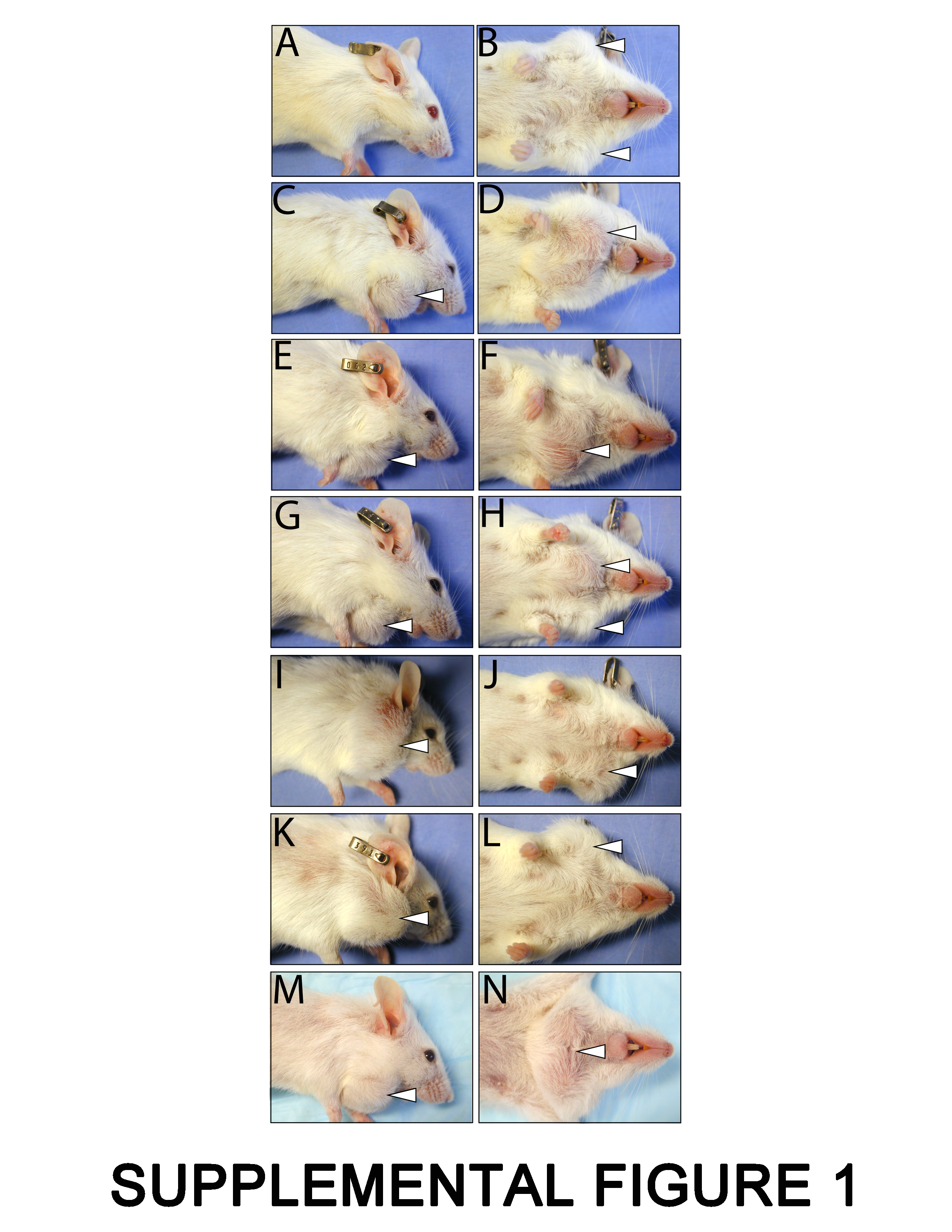

Supplement: S1 Fig — (A) is a lateral view of an age-matched WT mouse; (B-N) are representative images of lateral and ventral views of TG mice showing palpable salivary gland tumors (white arrowhead). Palpable tumors were detected in the parotid (B; H; J; and L), submandibular (D; F; H; and N), or both salivary gland types (B and H). (TIF) [file pone.0128467.s001.tif]

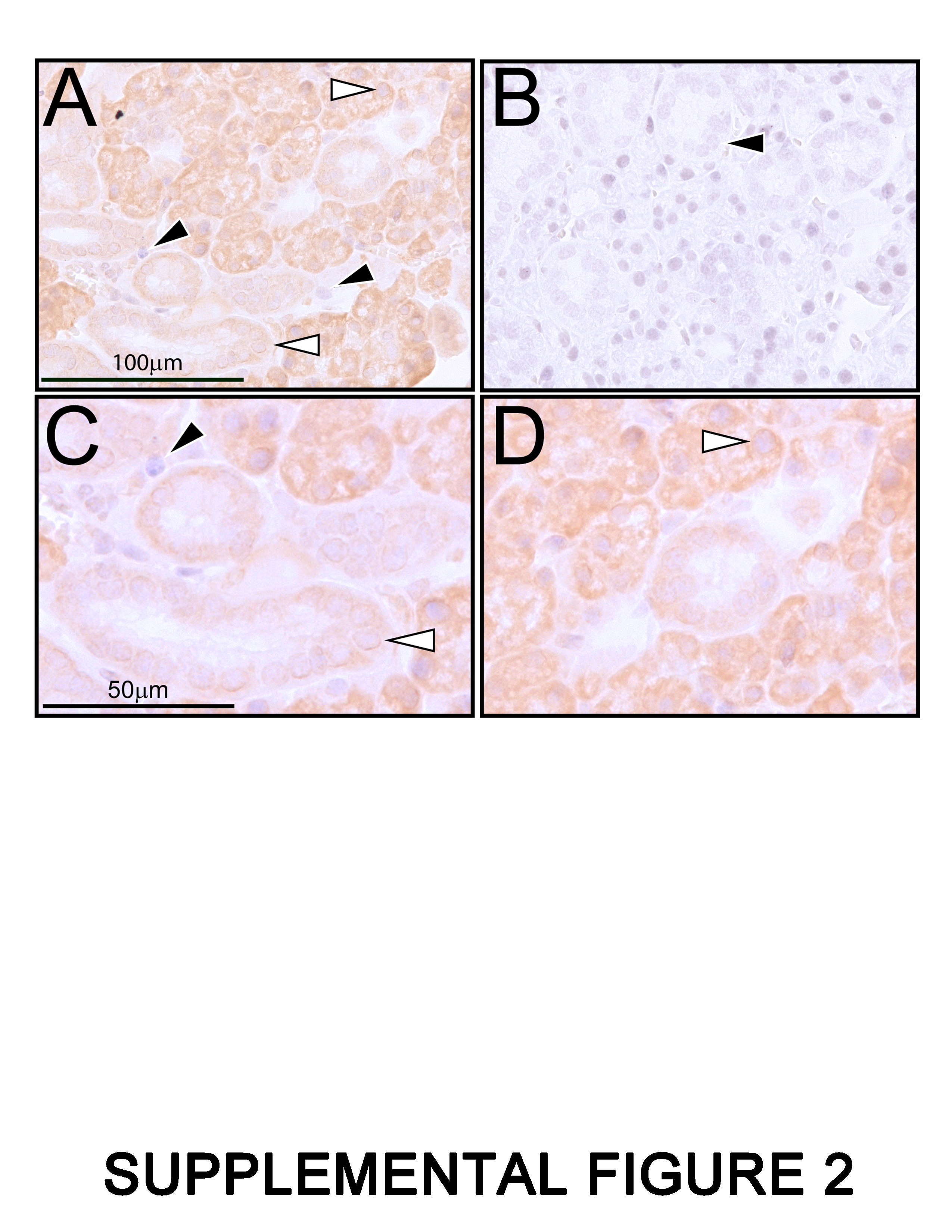

Supplement: S2 Fig — (A) shows typical RANK expression in the epithelium of salivary gland ducts and acini (white arrowheads); salivary gland cells that are negative for RANK expression are indicated by black arrowheads. (B) shows no immunoreactive staining in the absence of the primary antibody to RANK; scale bar in (A) also applies to (B). (C) and (D) are higher magnification images of regions shown in (A); scale bar in (C) also applies to (D). (TIF) [file pone.0128467.s002.tif]

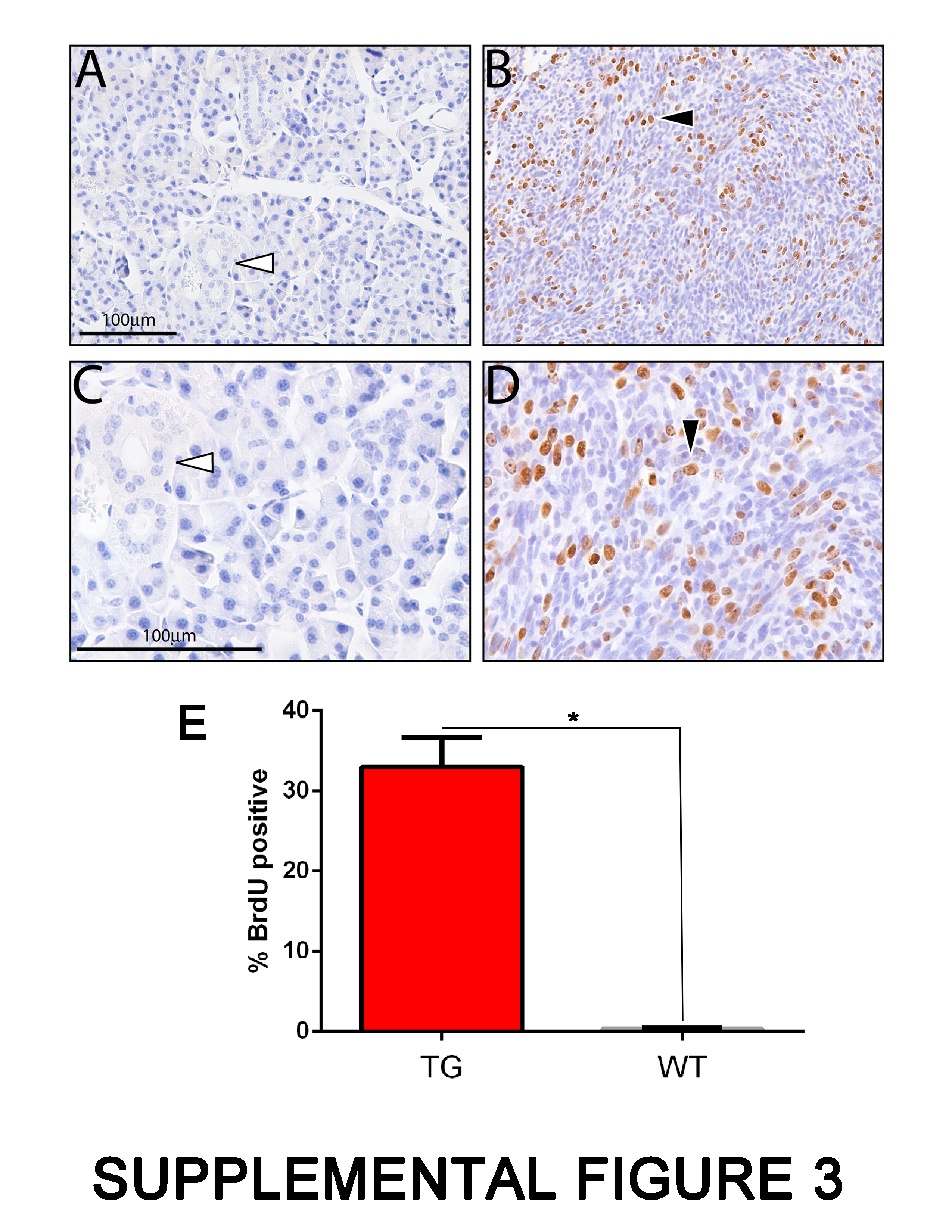

Supplement: S3 Fig — (A) and (C) are low and high magnification images respectively of salivary gland tissue from WT mice stained for BrdU incorporation; white arrowheads point to salivary gland ducts. (B) and (D) are low and high magnification images respectively of salivary gland tumor tissue from age-matched TG mice similarly stained for BrdU incorporation; black arrowheads point to BrdU positive tumor cells. Scale bar in (A) and (C) apply to (B) and (D) respectively. (E) Histogram displaying the average percentage of epithelial cells (± standard deviation (S.D.)) that are positive for BrdU incorporation in salivary gland tumors and salivary gland tissues of TG and WT mice respectively (*denotes p<0.05 (n = 4 mice/genotype)). (TIF) [file pone.0128467.s003.tif]

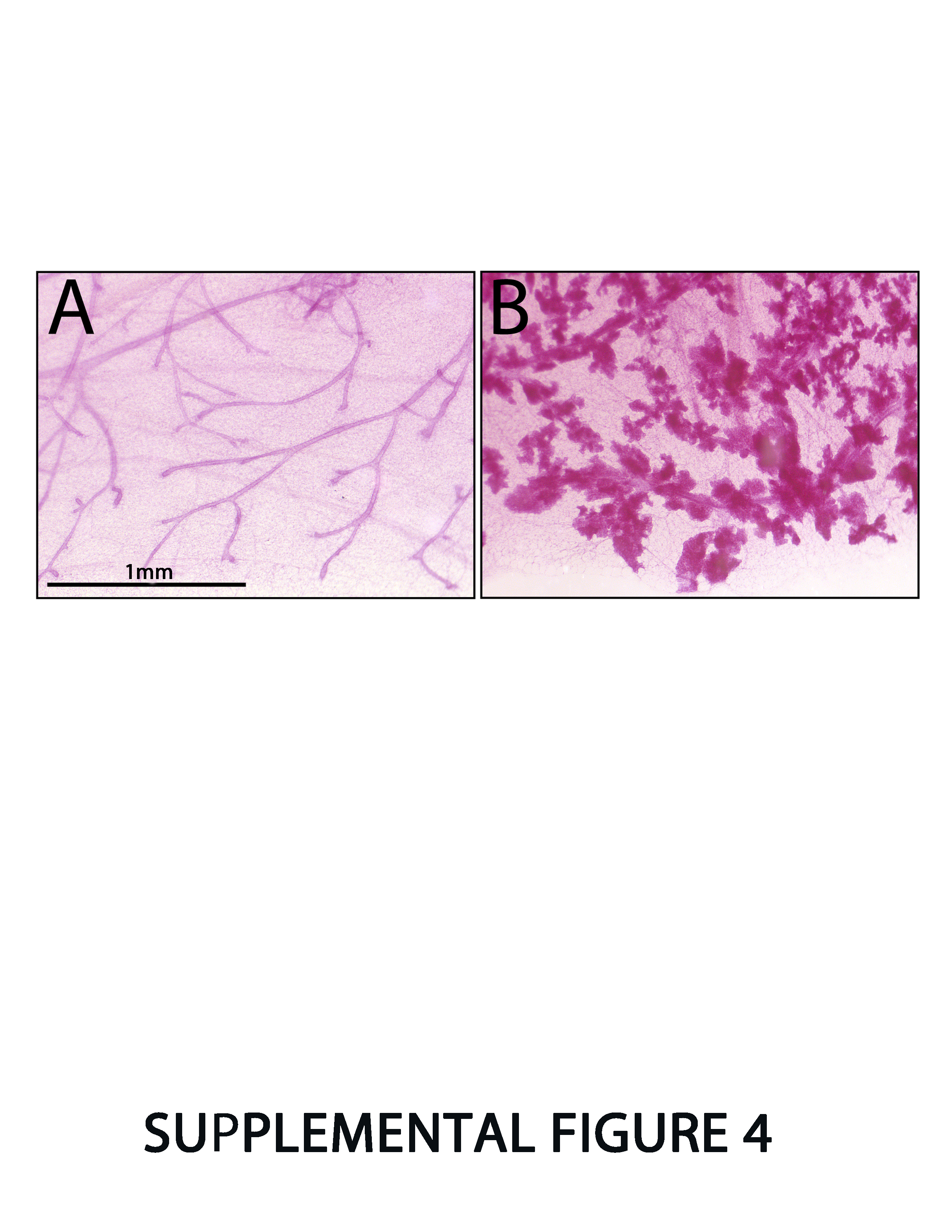

Supplement: S4 Fig — (A) and (B) are high magnification images of mammary gland whole mounts from age-matched WT and TG mice respectively. (B) Note the extensive ductal side-branching and alveologenesis in the TG mammary gland. Scale bar in (A) applies to (B). (TIF) [file pone.0128467.s004.tif]

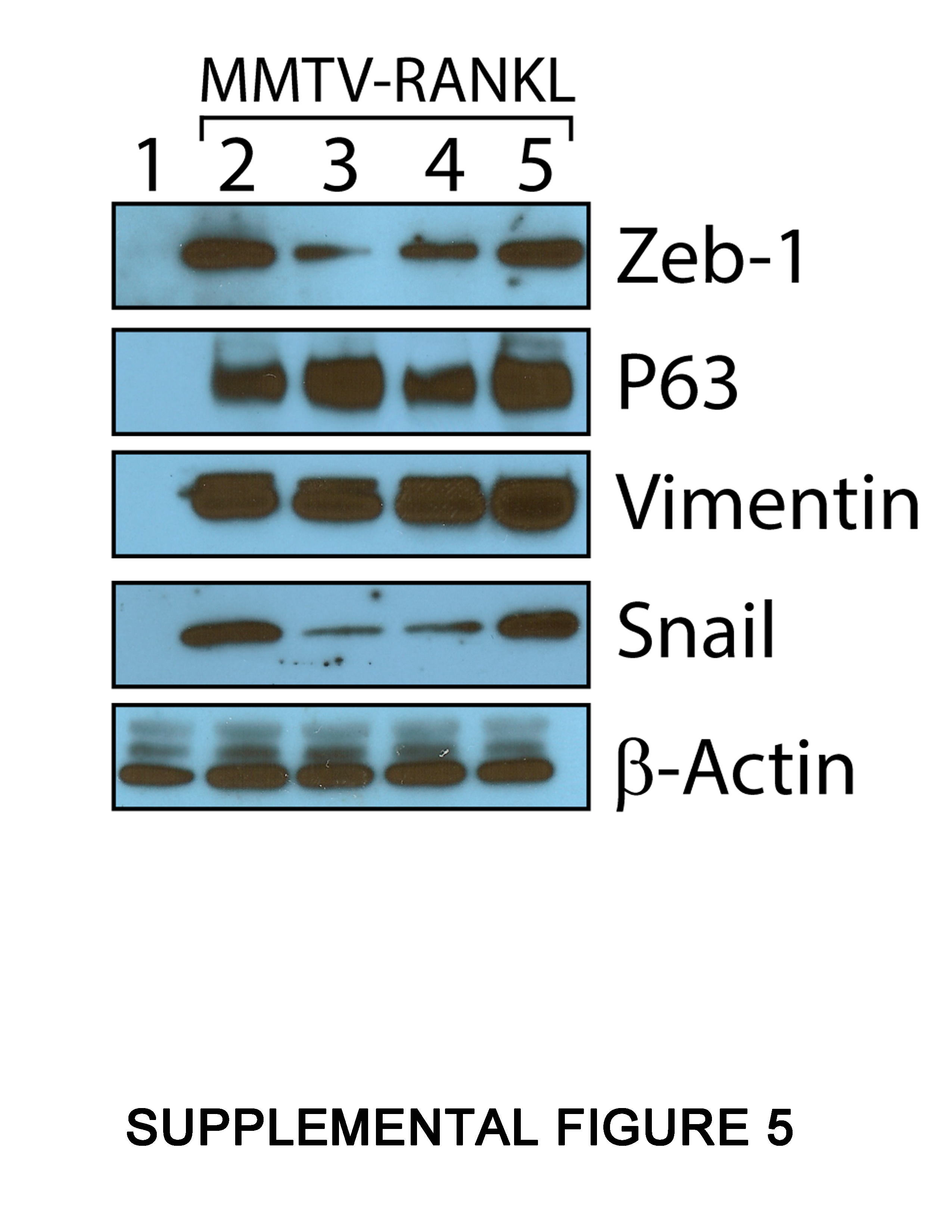

Supplement: S5 Fig — Western immunoblot result of salivary gland and salivary gland tumor protein isolated from WT mice (lane 1) and from four TG mice (lanes 2–5) respectively. Note the striking elevated levels of Zeb1, p63, vimentin, and snail/slug which represent common EMT molecular traits of an aggressive cancer phenotype. (TIF) [file pone.0128467.s005.tif]

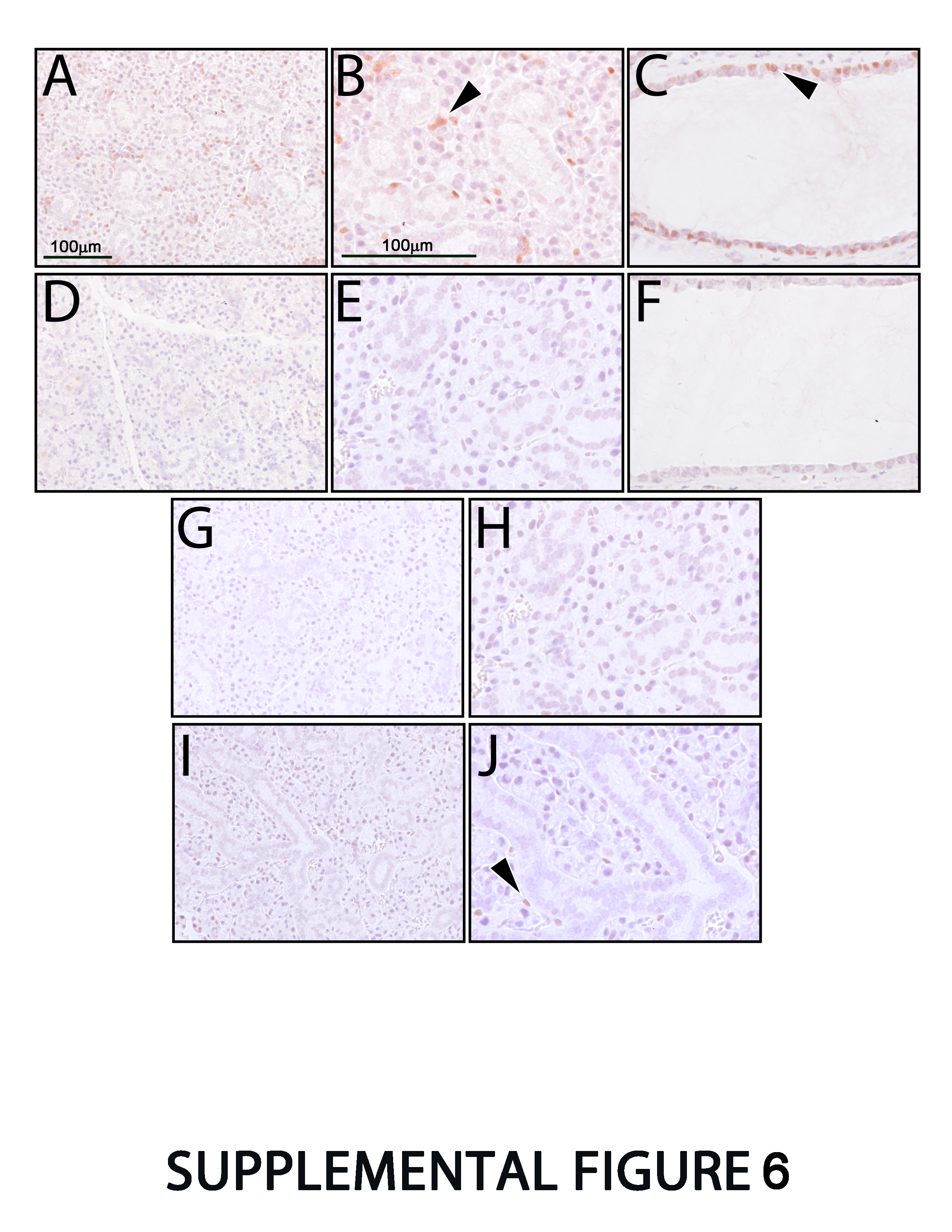

Supplement: S6 Fig — (A-C) are low and high magnification images of immunohistochemical detection of low levels of p63 expression in a subset of basal cells of the salivary gland epithelium of WT mice (black arrowhead). Snail/slug (D-F) and vimentin (G and H) are not detected whereas low levels of zeb1 expression are detected in a few cells per field (black arrowhead). Scale bar in (A) also applies to (D), (G), and (I); scale bar in (B) also applies to (C), (E), (F), (H) and (J). (TIF) [file pone.0128467.s006.tif]

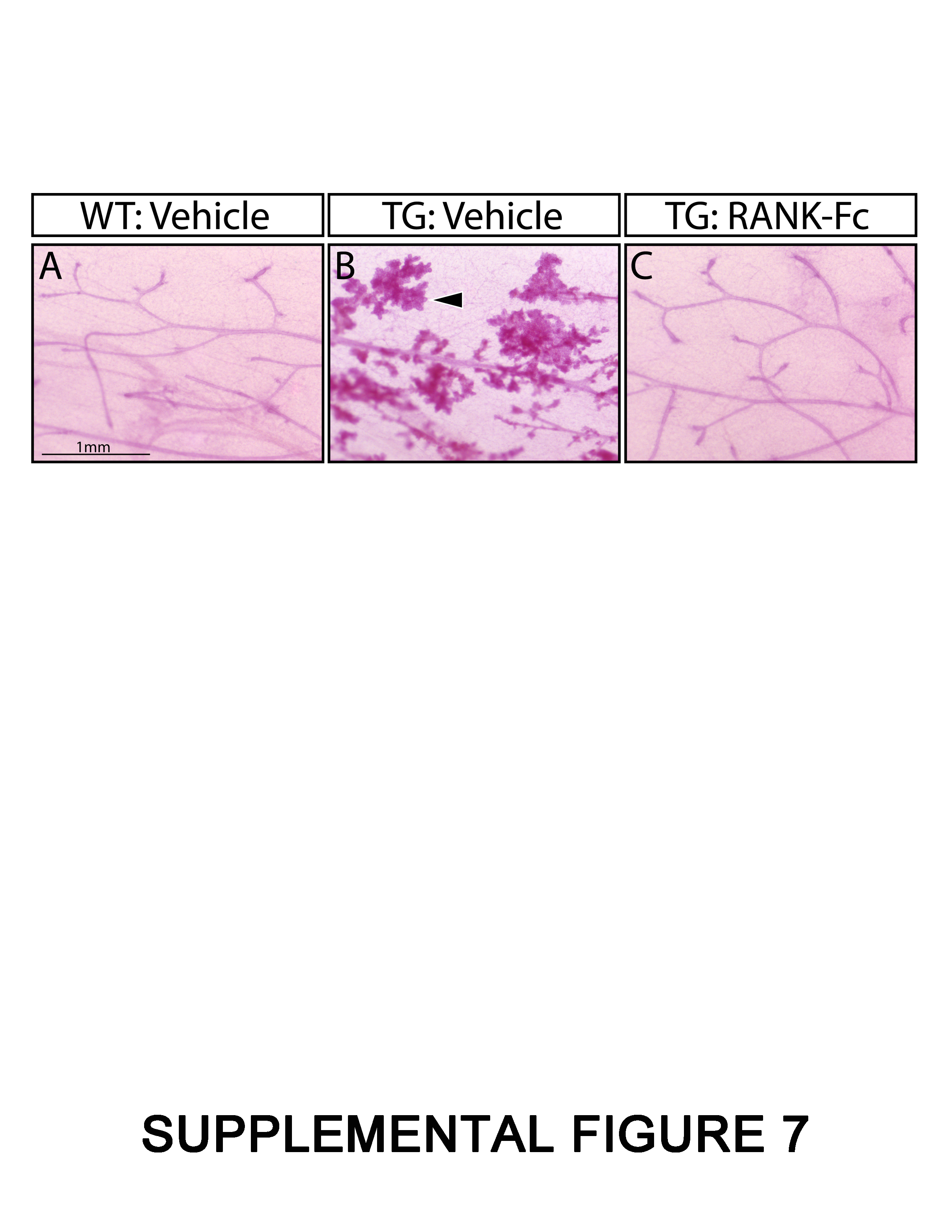

Supplement: S7 Fig — (A) Whole mount of mammary gland derived from a WT mouse treated with vehicle (PBS); see Fig 4 for full details of treatment protocol. (B) Whole mount of mammary gland from a TG mouse treated with vehicle. Note: clear evidence of epithelial alveologenesis driven by transgene-derived RANKL (black arrowhead). (C) Whole mount of mammary gland from a TG mouse following treatment with RANK-Fc. Note the absence of alveologenesis with RANK-Fc treatment. Scale bar in panel (A) applies to all panels. (TIF) [file pone.0128467.s007.tif]
