# Supplementary material for: Aberrant Activation of the RANK Signaling Receptor Induces Murine Salivary Gland Tumors
Source: PLoS One. 2015 Jun 10;10(6):e0128467. doi: 10.1371/journal.pone.0128467 (PMC4464738; doi:10.1371/journal.pone.0128467)

RANKL WESTERN

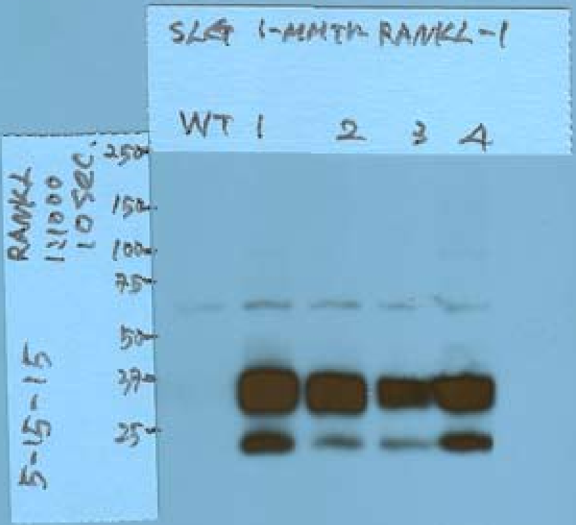

CYCLIN D1 WESTERN

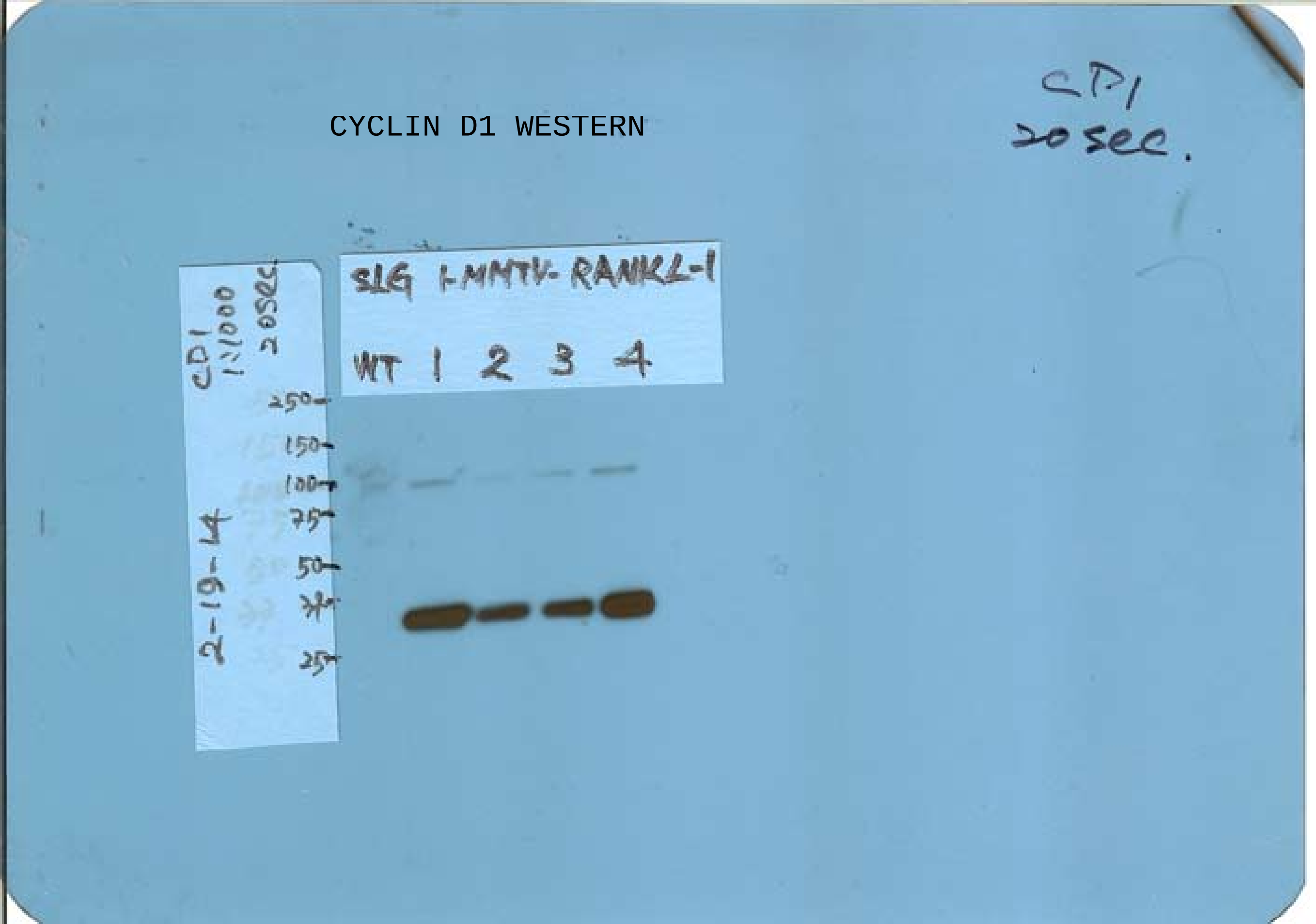

PCNA

PCNA WESTERN

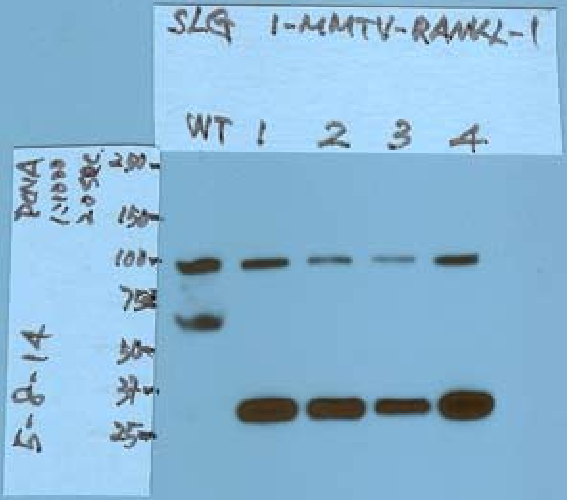

BETA-ACTIN WESTERN

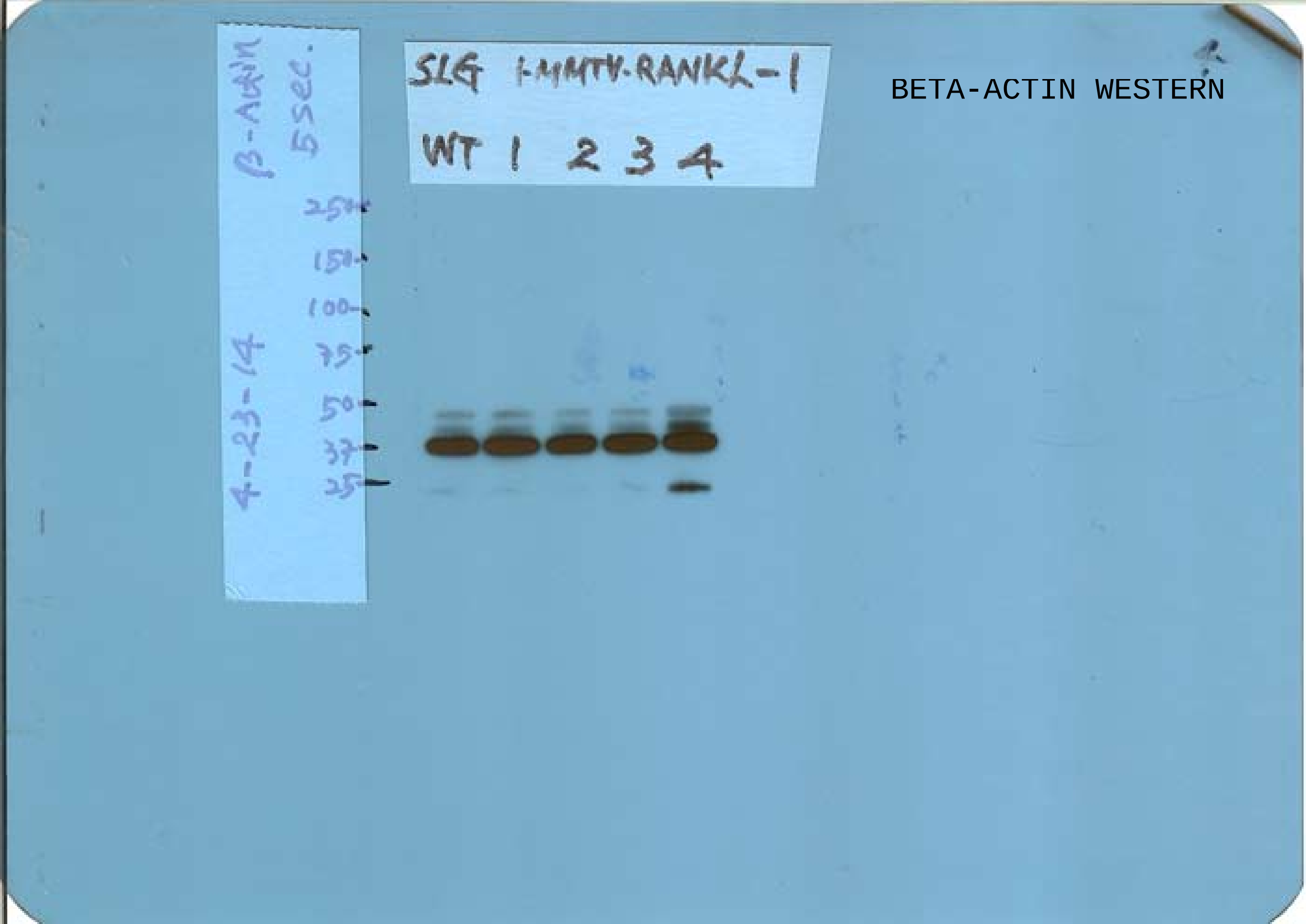

Supplement: S1 File — (PDF) [file pone.0128467.s008.pdf]
