# Supplementary material for: Aberrant Activation of the RANK Signaling Receptor Induces Murine Salivary Gland Tumors
Source: PLoS One. 2015 Jun 10;10(6):e0128467. doi: 10.1371/journal.pone.0128467 (PMC4464738; doi:10.1371/journal.pone.0128467)

ZEB-1 WESTERN

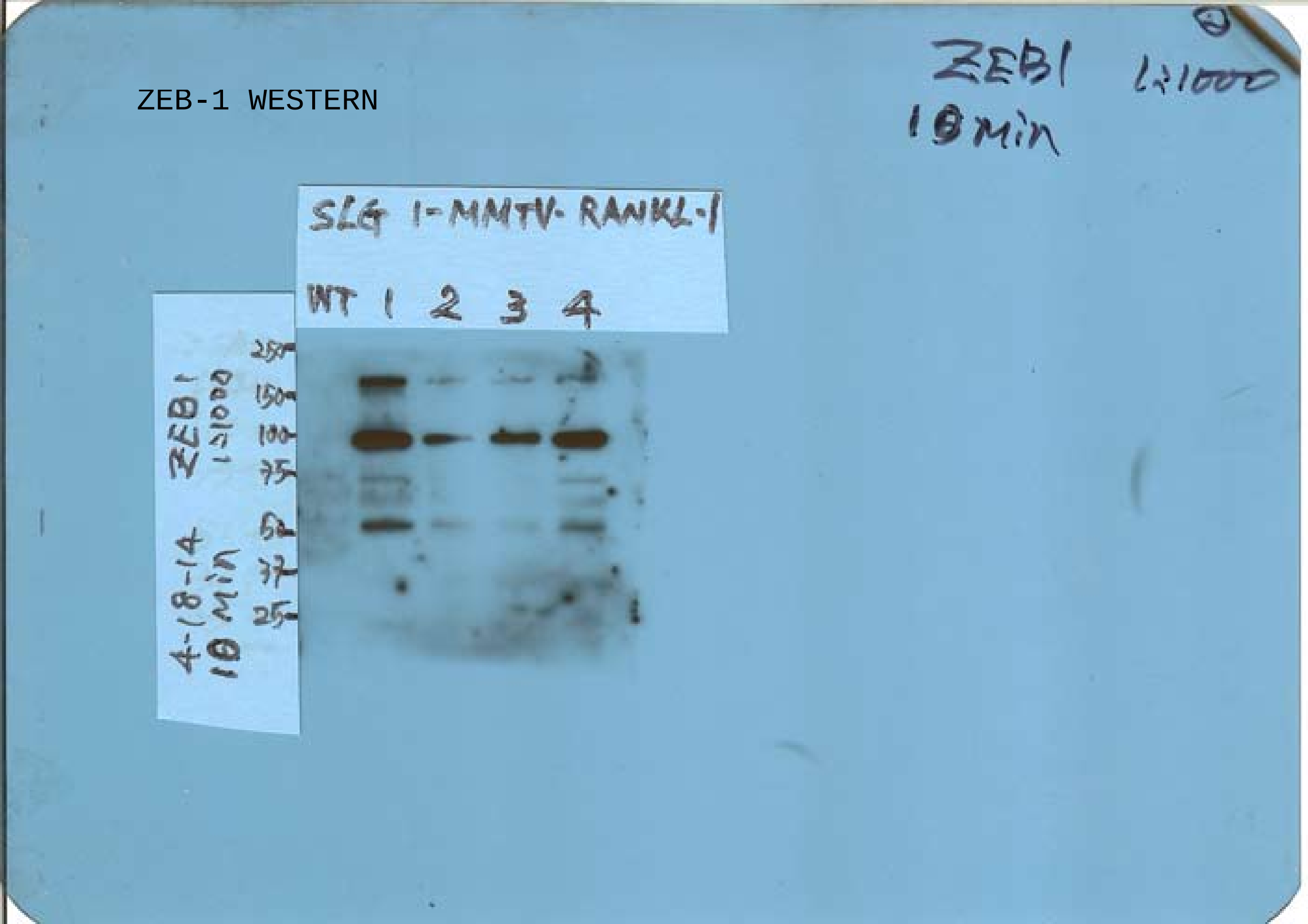

P63 WESTERN

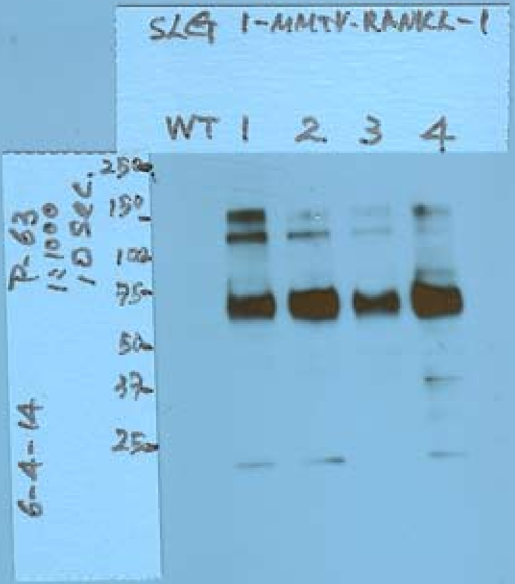
SLG 1-MMTV-RANKL-1
WT 1 2 3 4
250
150
100
75
50
37
25
P-63 1:1000 10 sec.
6-4-14

VIMENTIN WESTERN

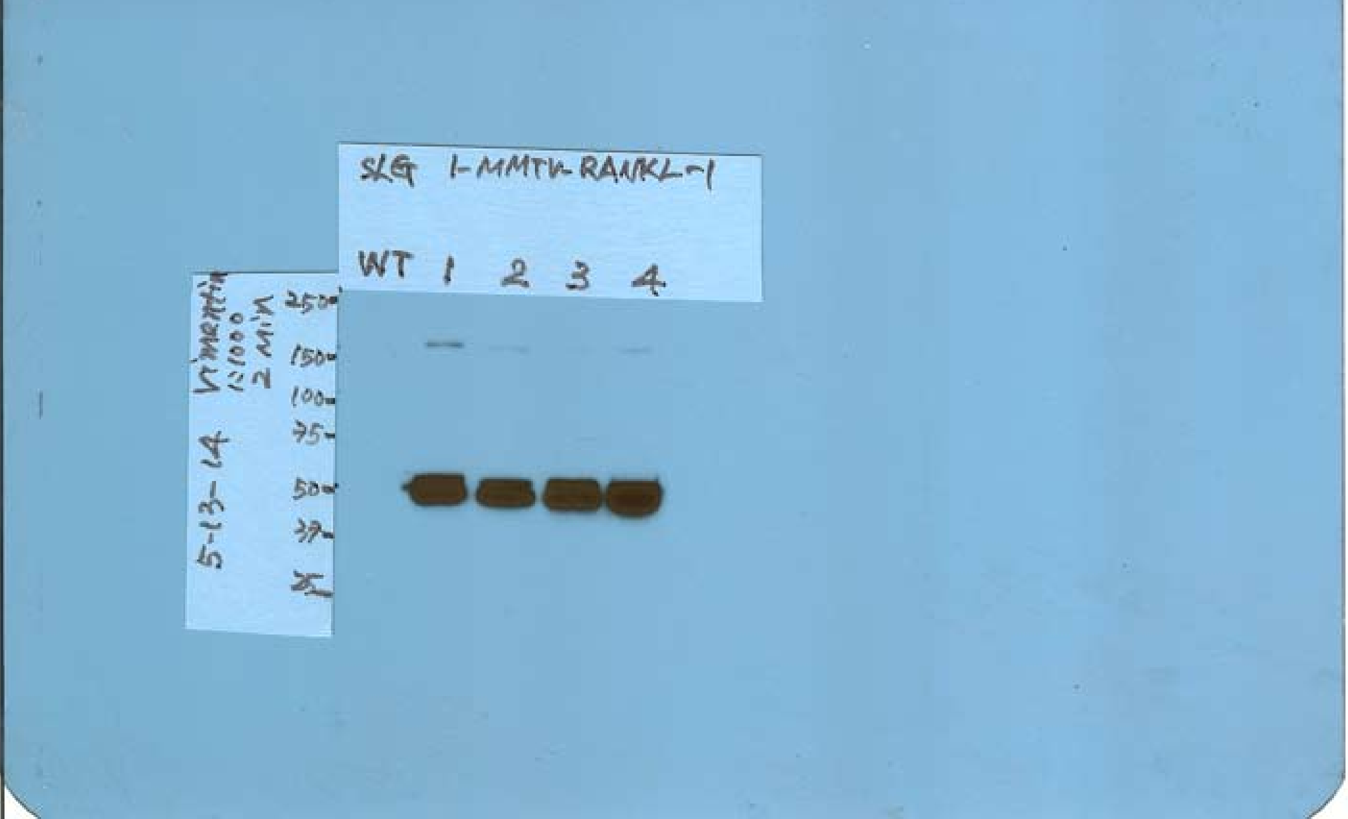

SNAIL SLUG WESTERN

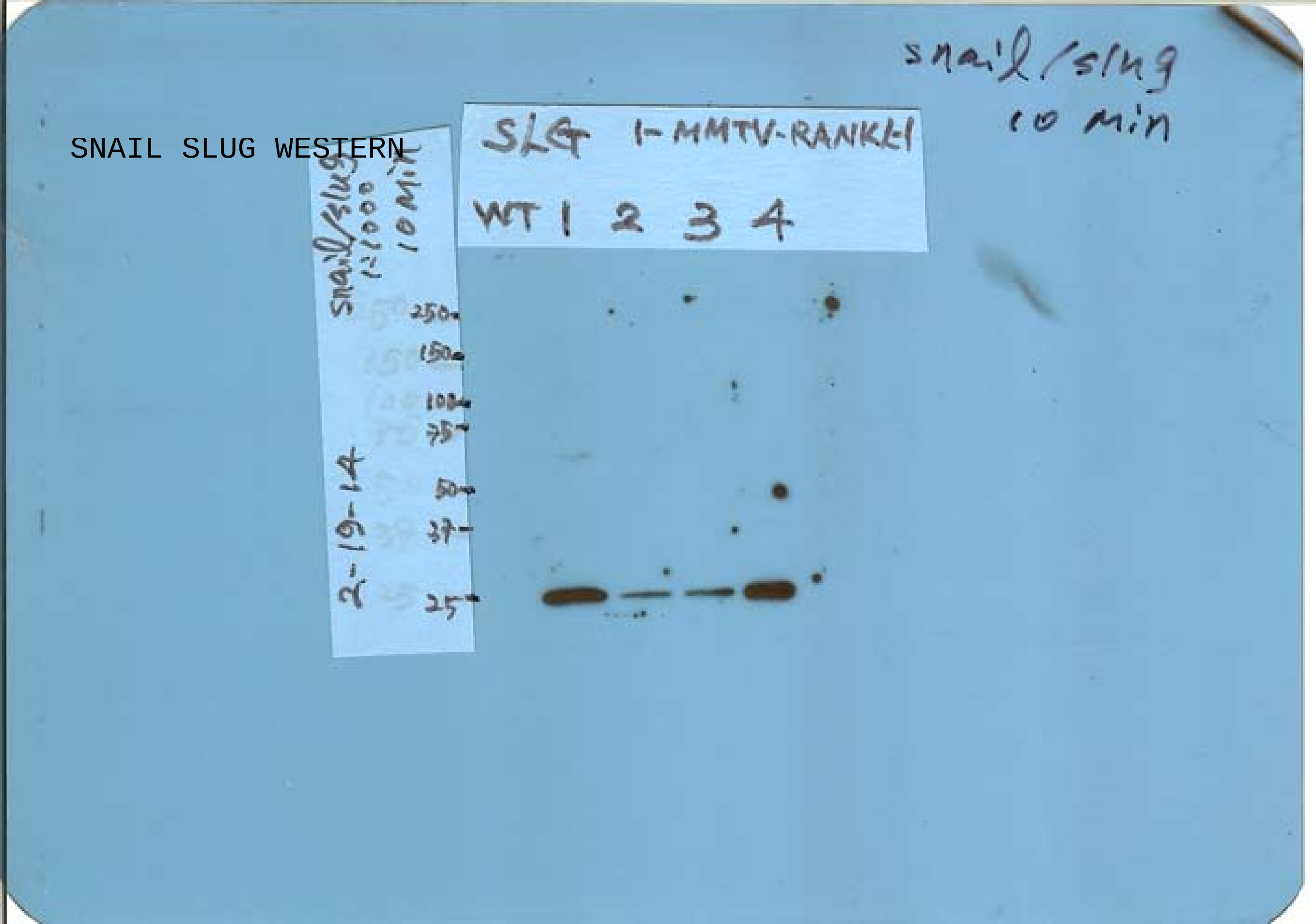

BETA-ACTIN WESTERN

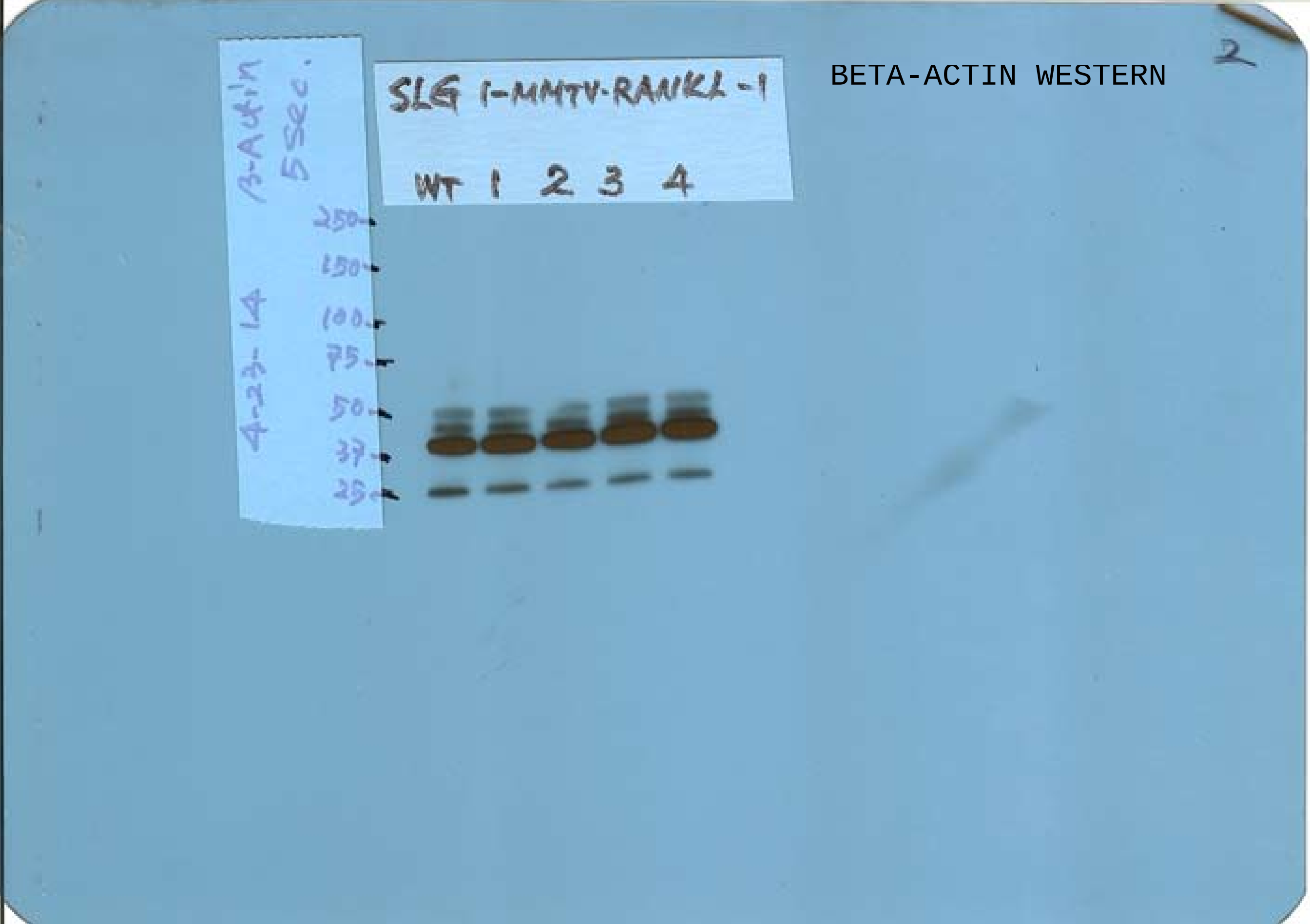

Supplement: S2 File — (PDF) [file pone.0128467.s009.pdf]
